# An advanced white matter tract analysis in frontotemporal dementia and early-onset Alzheimer’s disease

**DOI:** 10.1007/s11682-015-9458-5

**Published:** 2015-10-29

**Authors:** Madelaine Daianu, Mario F. Mendez, Vatche G. Baboyan, Yan Jin, Rebecca J. Melrose, Elvira E. Jimenez, Paul M. Thompson

**Affiliations:** 1 Imaging Genetics Center, Mark & Mary Stevens Institute for Neuroimaging & Informatics, University of Southern California, Marina del Rey, CA USA; 2 Department of Neurology, UCLA School of Medicine, Los Angeles, CA USA; 3 Behavioral Neurology Program, Department of Neurology, UCLA, Los Angeles, CA USA; 4 Departments of Neurology, Psychiatry, Radiology, Engineering, Pediatrics, and Ophthalmology, University of Southern California, Los Angeles, CA USA; 5 Brain, Behavior, and Aging Research Center, VA Greater Los Angeles Healthcare System, Los Angeles, CA USA; 6 Departments of Psychiatry and Biobehavioral Sciences, UCLA School of Medicine, Los Angeles, CA USA

**Keywords:** Early-onset Alzheimer’s disease, Behavioral variant frontotemporal dementia, Diffusion tensor imaging (DTI), Tract analysis, White matter

## Abstract

Cortical and subcortical nuclei degenerate in the dementias, but less is known about changes in the white matter tracts that connect them. To better understand white matter changes in behavioral variant frontotemporal dementia (bvFTD) and early-onset Alzheimer’s disease (EOAD), we used a novel approach to extract full 3D profiles of fiber bundles from diffusion-weighted MRI (DWI) and map white matter abnormalities onto detailed models of each pathway. The result is a spatially complex picture of tract-by-tract microstructural changes. Our atlas of tracts for each disease consists of 21 anatomically clustered and recognizable white matter tracts generated from whole-brain tractography in 20 patients with bvFTD, 23 with age-matched EOAD, and 33 healthy elderly controls. To analyze the landscape of white matter abnormalities, we used a point-wise tract correspondence method along the 3D profiles of the tracts and quantified the pathway disruptions using common diffusion metrics – fractional anisotropy, mean, radial, and axial diffusivity. We tested the hypothesis that bvFTD and EOAD are associated with preferential degeneration in specific neural networks. We mapped axonal tract damage that was best detected with mean and radial diffusivity metrics, supporting our network hypothesis, highly statistically significant and more sensitive than widely studied fractional anisotropy reductions. From white matter diffusivity, we identified abnormalities in bvFTD in all 21 tracts of interest but especially in the bilateral uncinate fasciculus, frontal callosum, anterior thalamic radiations, cingulum bundles and left superior longitudinal fasciculus. This network of white matter alterations extends beyond the most commonly studied tracts, showing greater white matter abnormalities in bvFTD versus controls and EOAD patients. In EOAD, network alterations involved more posterior white matter – the parietal sector of the corpus callosum and parahipoccampal cingulum bilaterally. Widespread but distinctive white matter alterations are a key feature of the pathophysiology of these two forms of dementia.

**Electronic supplementary material:**

The online version of this article (doi:10.1007/s11682-015-9458-5) contains supplementary material, which is available to authorized users.

## Introduction

Neuroimaging studies reveal white matter microstructural alterations in patients with dementia (Gold et al. 2012; Mahoney et al. 2014), including vascular infarcts, myelin breakdown, and progressive loss of axonal connectivity. In several subtypes of dementia, such as frontotemporal dementia (FTD) and Alzheimer’s disease (AD), gray matter loss also occurs in a characteristic pattern that has been used to differentiate the dementia subtypes on MRI (Thompson et al. 2003, 2004; Apostolova et al. 2007; Frisoni et al. 2007). Even so, we know far less about microstructural changes that accompany these cortical changes, and whether they are regionally specific. Because of the interest in the source of behavioral symptoms, it is important to know which white matter tracts are affected in each classical subtype of dementia. Standard anatomical MRI provides insufficient contrast to distinguish white matter tracts and measure their integrity. Recently, diffusion MRI has been added to major neuroimaging initiatives to better understand changes in white matter integrity and connectivity (Li et al. 2013; Jin et al. 2015; Zhan et al. 2015).

The two most common forms of neurodegenerative dementia are AD, followed by FTD. Under the unique rubric of AD fall early-onset and late-onset Alzheimer’s disease (EOAD and LOAD); both forms of AD present with the same neuropathological hallmarks (Frisoni et al. 2007; McKhann et al. 1984), involving amyloid plaques and neurofibrillary tangles, typically spreading from the entorhinal cortex, to the hippocampus and then to the rest of the cortex (Braak and Braak 1991); cortical gray matter is lost in a similar dynamic pattern (Frisoni et al. 2009). Here, we studied EOAD, which is less common than LOAD. Although the neuropathology of EOAD is consistent with the general neuropathology of LOAD, with neuritic plaques and neurofibrillary tangles, EOAD may have more parietal (precuneus and posterior cingulate) neurofibrillary tangles and hippocampal sparing on neuropathology. Furthermore, FTD, the second most common form of neurodegenerative dementia, is characterized by deficits in social conduct, emotion and insight (Seeley 2008) (Mendez and Shapira 2011). The neuropathology of FTD is usually characterized by frontotemporal atrophy and microvascular degeneration with intraneuronal inclusion bodies containing abnormal protein deposits, most commonly hyperphosphorylated tau or TDP-43. FTD includes a group of progressive degenerative syndromes among which behavioral variant frontotemporal dementia (bvFTD), also studied here, is most prevalent accounting for about 70 % of FTD cases (Pan and Chen 2013). Unlike LOAD, bvFTD presents with behavioral symptoms in the early stages, reflecting frontal, temporal and insular degeneration (Seeley 2008; Lu et al. 2013; Pan and Chen 2013; Walterfang et al. 2014).

Neuroimaging can assist in diagnosing EOAD and bvFTD, and there is growing interest in understanding the progressive breakdown of brain networks in each type of dementia (Mahoney et al. 2014). Diffusion weighted imaging (DWI) is a variant of standard anatomical MRI that enables the study of such white matter tract profiles. DWI generates multiple metrics of white matter integrity based on water diffusion in the tissue microstructure, where water molecules diffuse more rapidly along the white matter bundles than perpendicular to them due to barriers imposed by axonal membrane and myelin sheets (Acosta-Cabronero et al. 2010). Unlike other methods, DWI allows the study of brain connectivity by non-invasively tracing connections in the living tissue. Using DWI, we can better understand the disconnecting lesions in relation to their clinical symptoms (Catani and ffytche 2005). The disconnection paradigm – advanced by Geschwind in 1965 (Geschwind 1965a, b), has been proposed to partially explain AD symptomatology (Morrison et al. 1986; Morris 1996; Delbeuck et al. 2003). In this model, the symptoms of AD, including EOAD, are considered to be a consequence of the disturbance of brain’s effective connectivity (Daianu et al. 2015c), characterized by loss of afferent and efferent connections of cortical areas linked to death of pyramidal neurons (Morrison et al. 1986) that support information transfer within and between hemispheres (Delbeuck et al. 2003). As of recently, bvFTD has also been perceived as a disconnection syndrome where changes in network connectivity, particularly fronto-limbic disconnections (Farb et al. 2013), are the most defining disease-related features (Schroeter et al. 2015).

There are significantly more neuroimaging studies, including studies of white matter integrity, that investigated bvFTD than there are studies focused on EOAD. To our knowledge, information on white matter alterations in EOAD is limited, especially in comparison with an age-matched group of dementia patients. For bvFTD, measures of white matter disease are thought to be more sensitive than those for gray matter disease. In particular, two studies show that tractrography and DTI measures are more sensitive in terms of bvFTD diagnosis than gray matter volumetrics, indicating decreased fractional anisotropy (explained in the Methods section) in the cingulum, anterior corpus callosum, and uncinate fasciculus (Powers et al. 2014; Santillo et al. 2013). Other studies show that changes in white matter diffusion correlate with gray matter atrophy in bvFTD (Avants et al. 2010; Borroni et al. 2007; Whitwell et al. 2010).

Here we analyzed and visualized alterations in white matter fiber tracts in 20 bvFTD participants and 23 age-matched non-familial EOAD patients, compared to 33 healthy matched elderly participants. Unlike LOAD, EOAD is a form of AD with an early onset as seen in bvFTD patients, and frequent non-amnestic presentations involving language and visuospatial cognitive domains (Ishii et al. 2005; Frisoni et al. 2007; Karas et al. 2007). To detect commonalities and differences between the two forms of dementia, we used our newly developed method, autoMATE (automated multi-atlas tract extraction) (Jin et al. 2014). This method visualizes the full 3D profile of white matter bundles based on tractography (“fiber tracking”) (Jin et al. 2014) – not detectable by commonly used tract-based spatial statistical (TBSS) methods (Smith et al. 2007; Bodini et al. 2009). We hypothesized that with this method, we would find altered fiber integrity extending beyond tracts known to be affected in bvFTD – the uncinate fasciculus, cingulum bundle and corpus callosum (Mahoney et al. 2014). Similarly, in EOAD, where white matter alterations are less well-known, we aimed to define tracts that might contribute to the pathogenesis of the disease, in the ventral frontal cortex near the inferior fronto-occipital fasciculs and uncinate (Bendlin et al. 2010; Gold et al. 2012), and the parahippocampal cingulum. Our findings describe how tract deficits may contribute to the progressive degeneration of brain networks in each dementia subtype.

## Methods

### Participants and diffusion weighted brain imaging

Participants with bvFTD and with EOAD were recruited from an outpatient behavioral neurology clinic in an academic university medical center. BvFTD participants met criteria for “probable” bvFTD based on revised International Consensus Criteria (Rascovsky et al. 2011) by history reported by caregivers and findings on neuroimaging. Participants with EOAD were diagnosed according to the National Institute of Aging-Alzheimer’s criteria for clinically probable AD (G. M. McKhann et al. 2011). Given the usual presenile onset of bvFTD, in order to have age-matched groups, only EOAD patients with early-onset disease (<65 years of age) were included in the comparison group. Most of the EOAD patients had primarily an amnestic presentation and were non-familial, i.e., there was no history of autosomal dominant transmission or affected family members with EOAD; only 4 patients were visual variant and 5 were language variant. But all EOAD patients met criteria for EOAD. Moreover, clinically, confirmation of EOAD was made by cerebrospinal fluid analysis of low β42-amyloid and high total *tau* and phospho-*tau*. None of the EOAD patients had a previous history of a psychiatric disorder or neurological disease and none was currently taking medications that could impact performance on the neurologic exam. In addition, there were no bvFTD or EOAD patients with clinical or neuroimaging evidence of co-morbid neurological disorders such as dementia with Lewy bodies. PET imaging with florbetapir or other amyloid-binding radioligands was not available at the date of most of the MRI scan acquisitions. Across all three groups, individuals with major medical illnesses (except hypertension or diabetes) were excluded.

We analyzed DWIs from 20 bvFTD participants (60.7 ± 10.7 SD), 23 age-matched, non-familial EOAD participants (59.0 ± 5.0 SD) and 33 healthy controls (59.4 ± 0.9 SD) (Table 1). All 76 participants underwent whole-brain MRI on 1.5-Tesla Siemens Avanto scanners, at the MRI Center at UCLA. We collected standard anatomical T1-weighted sequences (256 × 256 matrix; voxel size = 1 × 1 × 1 mm^3^; TI = 900 ms; TR = 2000 ms; TE = 2.89 ms; flip angle = 40°), and DWIs using a single-shot multisection spin-echo echo-planar pulse sequence (144 × 144 matrix; voxel size: 2 × 2 × 3 mm^3^; TR = 9800 ms; TE = 97 ms; flip angle = 90°; scan time = 5 min 38 s). 31 separate images were acquired for each DTI scan: 1 T2-weighted image with no diffusion sensitization (a *b*_*0*_ image) and 30 diffusion weighted images (*b* = 1000 s/mm^2^).

**Table 1:** Demographic information for the 33 healthy controls, 20 bvFTD and 23 EOAD patients studied here. The mean age, breakdown by sex and Mini Mental State Examination (MMSE) scores are listed for each diagnostic group

	Controls	bvFTD	EOAD	Total
Age	59.4 ± 9.6 SD	60.7 ± 10.7 SD	59.0 ± 5.0 SD	59.6 ± 8.8 SD
Sex	14M/19F	8M/12F	10M/13F	32M/44F
MMSE	29.1 ± 0.9 SD	24.1 ± 4.7 SD	23.4 ± 4.2 SD	26.0 ± 4.3 SD

### Diffusion tensor imaging

Basser and colleagues introduced the concept of diffusion tensor imaging (DTI) to characterize the diffusion of water molecules as a three-dimensional ellipsoid (Basser et al. 1994). This can be decomposed into three principal directions of diffusion (eigenvectors), and three eigenvalues, which denote the relative amount of diffusion in each direction. The first eigenvector, *λ*_*1*_ (also referred to as axial diffusivity, AX), describes the direction of maximal ‘apparent’ diffusion, while the second and third eigenvectors along the two perpendicular directions, *λ*_*2*_ and *λ*_*3*_, are embedded in the plane orthogonal to the main diffusion (Basser et al. 1994). Average diffusivity, inferred from the three eigenvectors of the diffusion ellipsoid, is known as mean diffusivity (MD), $MD=\frac{\lambda_1+{\lambda}_2+{\lambda}_3}{3}$. Moreover, the average of *λ*_*2*_ and *λ*_*3*_ defines radial diffusivity (RD): $RD=\frac{\lambda_2+{\lambda}_3}{3}$; when disrupted, it can indicate demyelination (Klawiter et al. 2011) if corresponding eigenvectors properly align with underlying tissue microstructure (Klawiter et al. 2011). Finally, the most widely studied DTI metric is fractional anisotropy: $FA=\sqrt{\frac{3}{2}}\frac{\sqrt{{\left({\lambda}_1-MD\right)}^2+{\left({\lambda}_2-MD\right)}^2+{\left({\lambda}_3-MD\right)}^2}}{\sqrt{\lambda_1^2+{\lambda}_2^2+{\lambda}_3^2}}$. FA has been the most cited DWI metric in studies of neurodegeneration (Acosta-Cabronero et al. 2010; Mahoney et al. 2014), and it can help to analyse its component eigenvalues, to better understand white matter changes. Acosta-Cabronero and colleagues hypothesized that proportional changes in AX and RD would keep FA relatively unchanged, as FA depends on this ratio. Changes in AX, RD and MD had greater effect sizes than FA reductions in the white matter of AD patients (Acosta-Cabronero et al. 2010), which we also found in our studies of participants with mild cognitive impairment (MCI) and AD (Nir et al. 2013).

### Image processing and tractography

DWIs were skull stripped with FSL’s function ‘BET’ and eddy corrected with the ‘eddy_correct’ function (http://fsl.fmrib.ox.ac.uk/fsl/) as done in (Daianu et al. 2012, 2013, 2015a). Then, tensor reconstruction was performed on these pre-processed images using the standard diffusion tensor imaging (DTI) scheme in Camino (http://cmic.cs.ucl.ac.uk/camino/). Fiber tracing was run using the probabilistic algorithm called the ‘Probabilistic Index of Connectivity’ (PICo). Seed points were chosen based on the FA maps at voxels with intensity values greater than 0.4 and Monte Carlo simulations were used to generate the fiber proceedings from these seed points (Jin et al. 2014). For each subject, ~40,000 fibers were traced at a maximum fiber turning angle of 60°, as suggested by recent studies (Thomas et al. 2014) to optimize specificity and sensitivity in DTI. Fiber tracing stopped at a commonly used FA threshold lower than 0.2. These initial processing steps were implemented in a large workflow, known as autoMATE (automated multi-atlas tract extraction), developed by our laboratory and fully described in (Jin et al. 2012, 2013, 2014).

### Fiber clustering and label fusion in autoMATE

For the next step in autoMATE, we defined five white matter representative tract “atlases” from 3 male and 2 female right-handed healthy participants based on the “Eve” brain template, which was parcellated into 130 bilateral regions of interest (ROIs) (Zhang et al. 2010; Jin et al. 2014). Participants were selected from our healthy control group. Our tract atlases consisted of 21 major bundles, listed in Table 2. To construct them, we first linearly and non-linearly registered the Eve template to each participant’s FA map using the Advanced Normalization Tool (ANTS) (Avants et al. 2011) that provided within-millimeter accuracy. Then, the Eve ROIs were transferred to each atlas with the deformation field generated by registration (Jin et al. 2014). We extracted 21 sets of tracts with the corresponding ROIs based on a look-up table (Zhang et al. 2010). The five tract atlases were manually edited and inspected by a neuroanatomically trained image analyst to ensure accuracy.

**Table 2:** Representative fiber bundles

#	ROI	Name^#fibers^
1	L-ATR	left anterior thalamic radiation^268^
2	R-ATR	right anterior thalamic radiation^289^
3	CC-FNR	corpus callosum, frontal^852^
4	CC-OCC	corpus callosum, occipital^335^
5	CC-PAR	corpus callosum, parietal^255^
6	CC-POCG	corpus callosum, post-central gyrus^114^
7	CC-PRCG	corpus callosum, pre-central gyrus^143^
8	CC-TEM	corpus callosum, temporal^178^
9	L-CGC	left cingulum^365^
10	R-CGC	right cingulum^326^
11	L-CST	left corticospinal tract^195^
12	R-CST	right corticospinal tract^274^
13	L-IFO	left inferior fronto-occipital fasciculus^377^
14	R-IFO	right inferior fronto-occipital fasciculus^309^
15	L-ILF	left inferior longitudinal fasciculus^353^
16	R-ILF	right inferior longitudinal fasciculus^340^
17	L-PHC	left parahippocampal cingulum^101^
18	R-PHC	right parahippocampal cingulum^155^
19	L-SLF	left superior longitudinal fasciculus^297^
20	L-UNC	left uncinate fasciculus^183^
21	R-UNC	right uncinate fasciculus^150^

The corpus callosum tracts are divided into 6 distinct segments: frontal, temporal, parietal, occipital, precentral gyrus and postcentral gyrus. The SLF tract was only traced in the left hemisphere as it tends to be highly asymmetrical in population studies (Catani et al. 2007). For each fiber tract, we indicate the total number of fibers included in the atlas template that we used for our population studies

Then, we registered the FA maps of those five atlases to the FA maps of the rest of the participants. The 21 sets of tracts of each atlas were warped to the DWI spaces of the remaining participants with the deformation fields obtained from the registration (Jin et al. 2011). Fiber extraction of each tract, as detailed in (Jin et al. 2014), was based on the Hausdorff distance between the tract from each atlas and the participant’s fiber candidates from the ROI extractions (Jin et al. 2014; Dennis et al. 2015a, b). Then, individual fiber bundles were fused from the 5 atlases and visually inspected for accuracy by the same image analyst.

Lower FA in brain regions with atrophy or lesions may make it harder to track fibers in patients with dementia. Unlike other methods, autoMATE does not restrict the tract-based analyses to areas where fibers are reconstructed. Instead, in areas with low FA (where fibers might not be traced), autoMATE uses the FA metric at that particular point in the registered FA map for the tract-based analyses. Therefore, the fiber maps provide a standardized space to conduct the analyses, which does not require complete detection of fiber tracts (Jin et al. 2014; Dennis et al. 2015a, b). Each fiber was uniformly sampled using 15 equidistant points; these were associated with DTI measures FA, MD, RD and AX.

### Statistical analyses

In a point-wise tract matching scheme method, part of autoMATE (Jin et al. 2014), we studied the full 3D profile of all 21 fibers as functions of FA, MD, RD and AX. The matching scheme incorporated by autoMATE guarantees point-wise correspondence for each tract across the population. We tested for group differences between each pair of diagnostic groups – bvFTD vs. controls, EOAD vs. controls and bvFTD vs. EOAD – by running a linear regression (i.e., coding disease status as 1 and controls as 0), controlling for age, sex and brain volume. We corrected for multiple comparisons using the False Discovery Rate (FDR) (Benjamini and Hochberg 1995) (*q* < 0.05). To explain further, for each DTI metric, we FDR corrected across all 87,885 tested data points that arose from analyzing a total of 5859 fibers (summed across all 21 tracts), each sampled at 15 distinct points (87,885 = 5859 × 15). Next, we assessed the effect size, *r*, across the entirety of each of the 21 tracts by incorporating the *t*-values, as output by the linear regression, and the degrees of freedom (df) – computed as the total number of participants within each test minus 2:

$$ r=\sqrt{\frac{t^2}{t^2+df}} $$

## Results

**Fig. 1:**
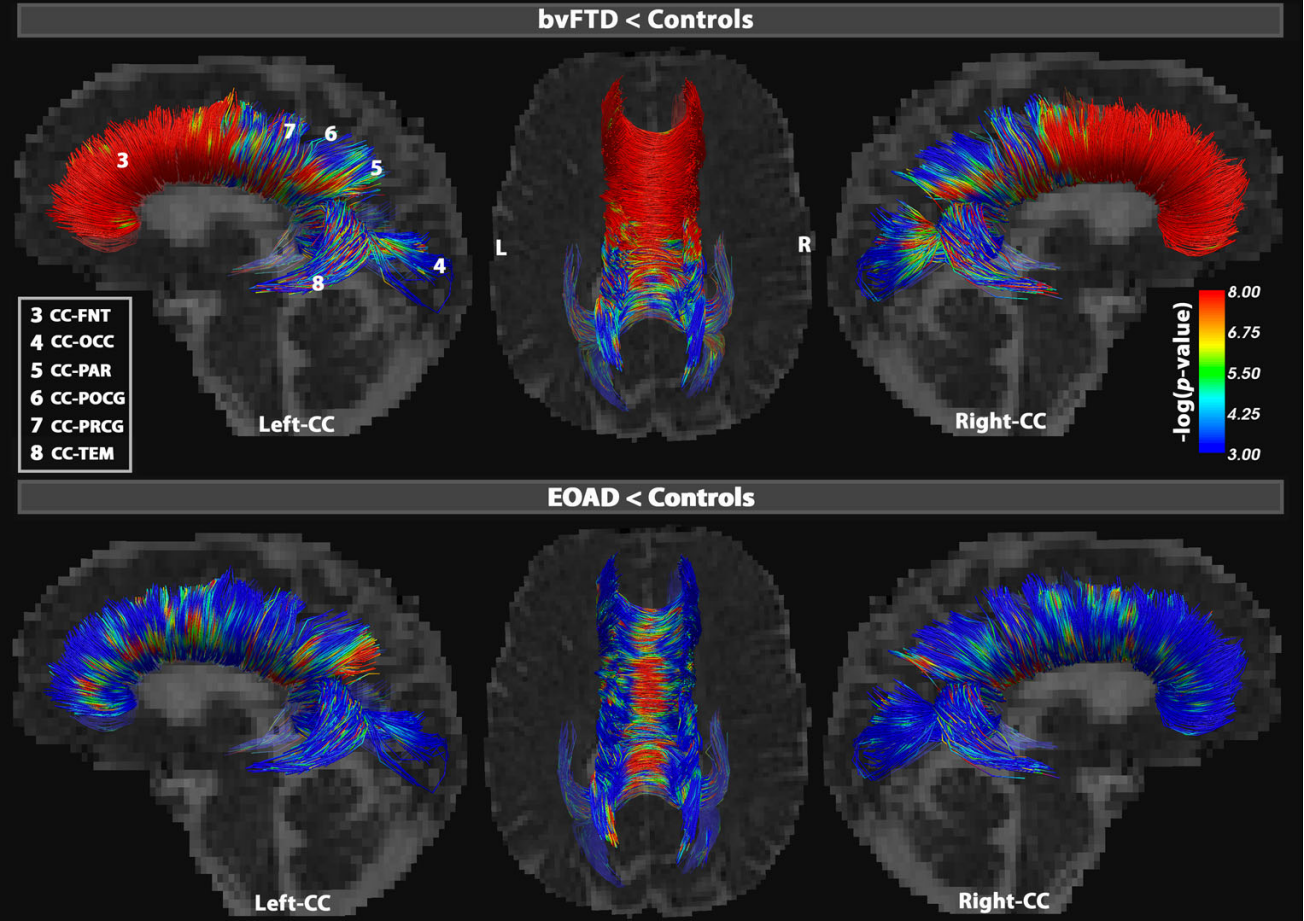
Point-wise tract differences in the segments of the corpus callosum (CC). Results illustrate increasing MD, the most sensitive DTI metric to detect differences between bvFTD and healthy controls (*upper figures*) and EOAD and healthy controls (*lower figures*). The CC was divided into 6 distinct segments: CC-FNT, CC-POCG, CC-PRCG, CC-PAR, CC-TEM and CC-OCC. In bvFTD, the CC-FNT showed 98 % MD alterations, while at least 35 % of the other segments were also significantly altered (FDR critical *P* = 0.03). In EOAD, MD detected differences among, but not restricted to, the lateral projections of the CC, especially in the CC-PAR where 43 % of fibers were disrupted, followed by 35 % in CC-TEM and 33 % in CC-FRN (FDR critical *P* = 0.01). Color bars describe -log *p*-values FDR corrected across all tested points (Table 2)

**Fig. 2:**
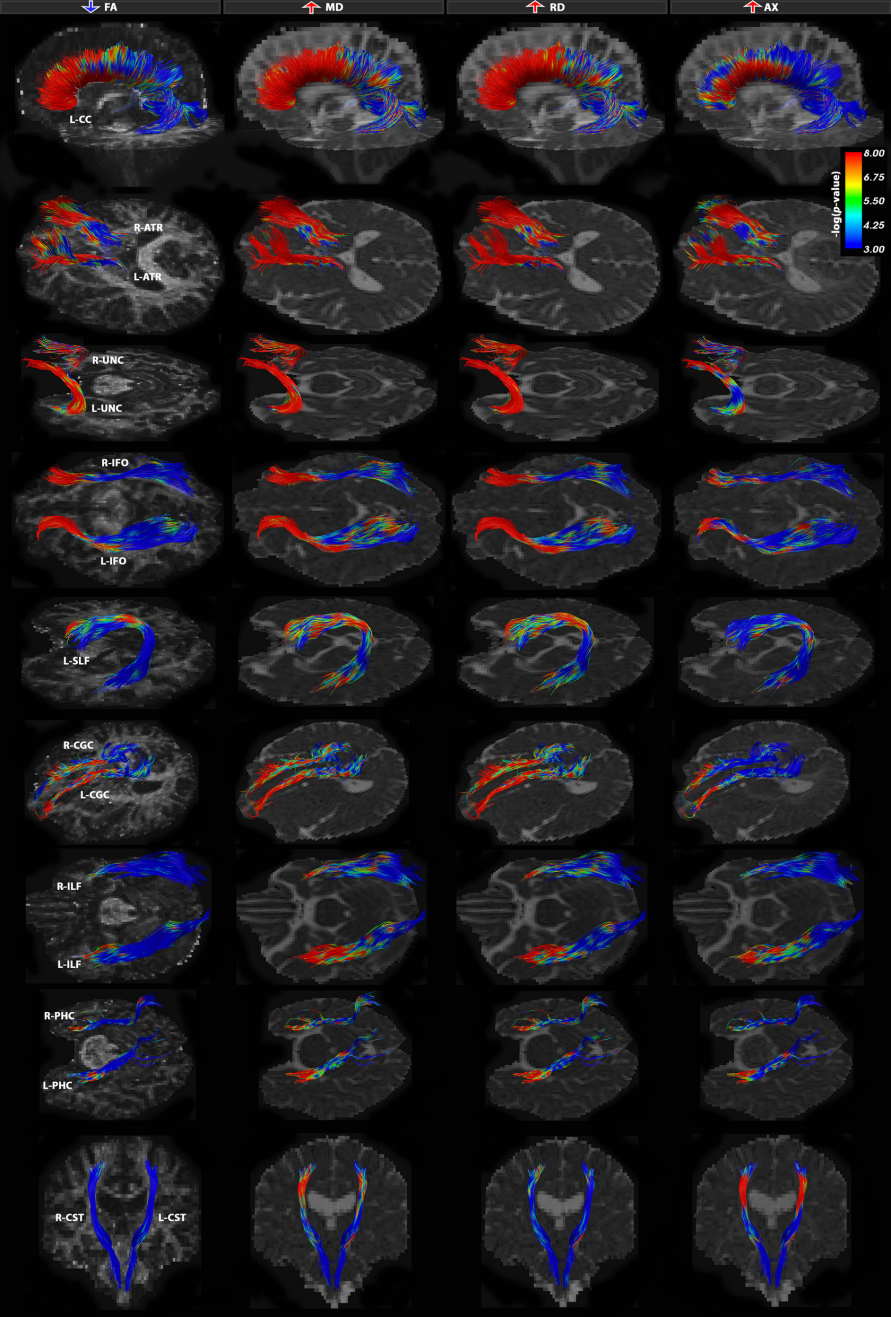
Tract-based analysis group differences between bvFTD and healthy controls for FA, MD, RD and AX DTI measures. All 21 bundles were severely affected in bvFTD patients with a specific neural network of alterations focused around the frontal and medial-frontal white matter connections. There were two major patterns of disruption – both involving simultaneous increases in measures RD and MD, with decreases in FA across the same set of fibers and/or increases in AX, suggestive of neurodegeneration. Tracts that were affected almost in their entirety (>80 % tracts) were the UNC bilaterally, CC-FNT and ATR fibers bilaterally. Color bars describe -log *p*-values FDR corrected across all tested points for each DTI measure (Table 3)

**Fig. 3:**
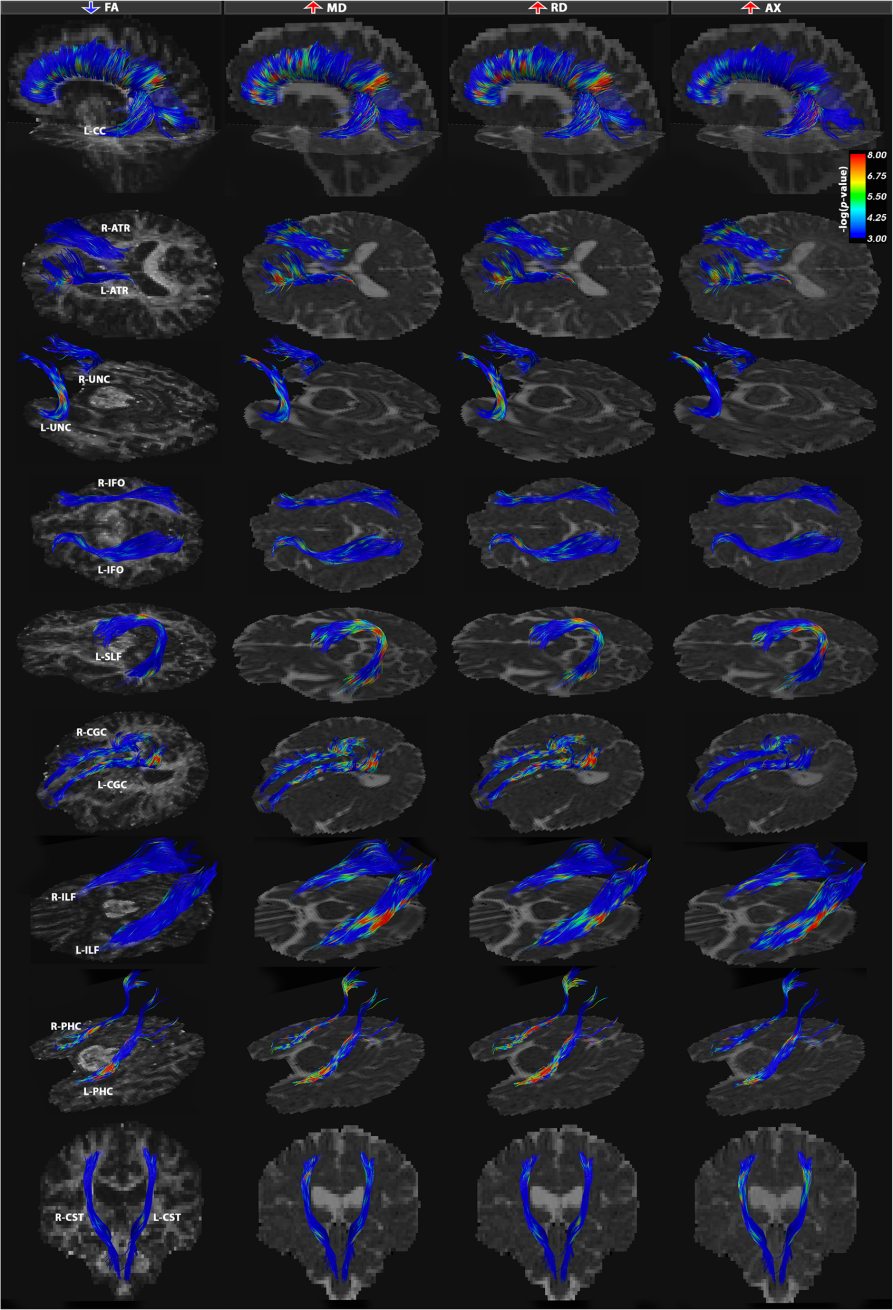
Tract-based analysis group differences between EOAD and healthy controls for FA, MD, RD and AX DTI measures. All 21 bundles were significantly affected in EOAD patients with a specific neural network of alterations focused in the posterior temporo-parietal network and caudal segments of the callosal fibers. Most characteristic patterns of disruption in the DTI measures indicate simultaneously increasing MD and RD measures with often increasing AX across overlapping fibers and a minimally affected or relatively unchanged FA. Most affected tracts were the PHC fibers bilaterally, CC-PAR and posterior segments of the cingulum bundle. Color bars describe -log *p*-values FDR corrected across all tested points for each DTI measure (Table 3)

**Fig. 4:**
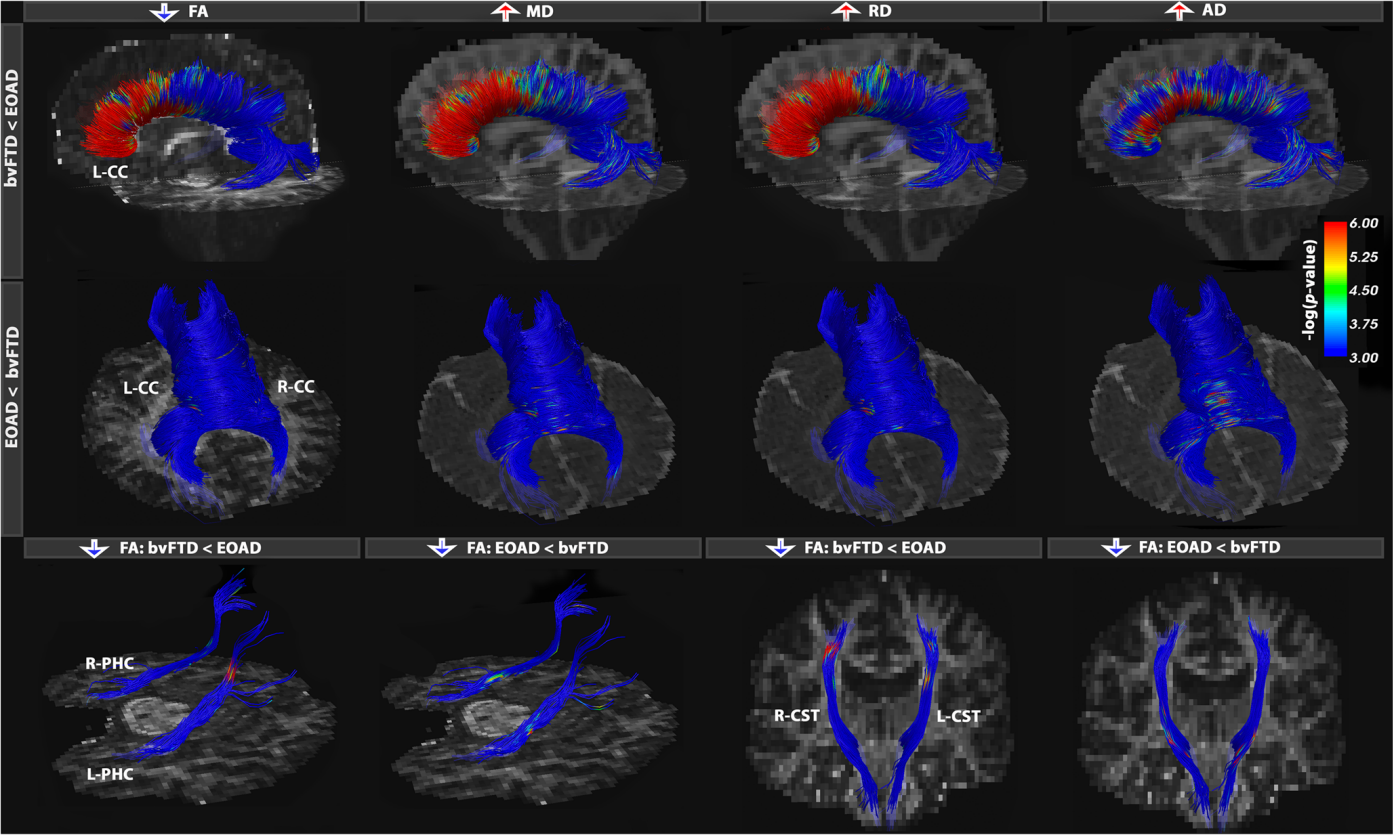
Tract-based analysis group differences between bvFTD and EOAD participants. Results show only fibers where differences were found in bvFTD vs. EOAD, as well as in EOAD vs. bvFTD. These results further emphasize the specific neural networks implicated in the neurodegeneration mechanisms in the two forms of dementia. In bvFTD, we note the involvement of an anterior network, while in EOAD, a more posterior network is most characteristic. Tracts with most alterations were the anterior and posterior segments of the CC, PHC and CST fibers. Color bars describe -log *p*-values FDR corrected across all tested points for each DTI measure (Online Resource 1)

Relative to healthy matched controls, fiber tracts were abnormal in both dementia groups (Figs. 1, 2, 3 and 4). Tracts connecting cortical regions with preferential atrophy showed greatest white matter alterations. Our spatially detailed tract analyses detected several alterations beyond those typically reported for each disease.

**Fig. 5:**
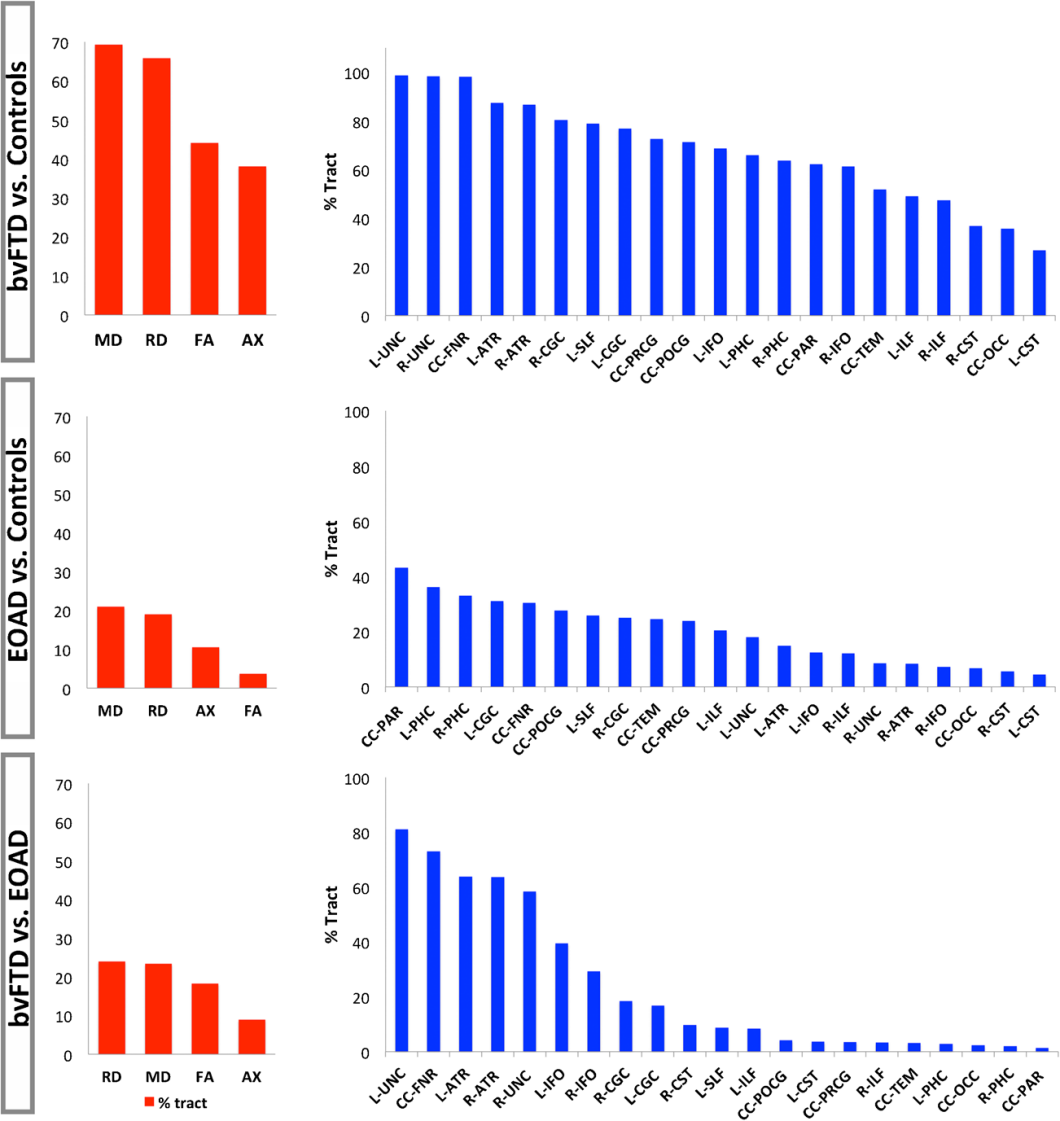
DTI measures ranked based on their ability to detect widespread white matter alterations. The % tract alterations as detected by each DTI measure, FA, MD, RD and AX, were averaged across all 21 bundles. Most alterations were detected by measures MD and RD in all group comparisons, patients vs. controls, and between patients (*red* bars). Next, % tract alterations from MD and RD were averaged and the fibers with greatest disruptions were ranked from high to low (*blue* bars). These rankings distinguish critical components of the neural networks involved in the degeneration mechanism in each form of dementia

Below we list the most prominent changes in DTI measures: decreases in FA, and increases in MD, RD and AX; these metrics are ranked in Fig. 5 based on the % of tracts with alterations in each of these common metrics. In most cases, these DTI tensor changes did not occur concurrently across the same fraction of fibers. As mentioned above, from a methodological standpoint – simultaneous and proportional increases in MD, RD and AX would leave FA relatively unchanged. If these changes are disproportional across widespread areas in the white matter, as we will see in bvFTD, FA changes too.

### BvFTD vs. controls

In our point-wise analyses, all 21 white matter bundles were severely affected in bvFTD patients, relative to controls, with higher MD (FDR critical *P* = 0.035) and RD (FDR critical *P* = 0.034). We also detected lower FA (FDR critical *P* = 0.024) and higher AX measures (FDR critical *P* = 0.019) but not in the exact same fibers (see Table 3; Figs. 1 and 2).

**Table 3:** Comparisons between dementia groups and healthy controls

#	ROI	bvFTD < CTL								EOAD < CTL							
		↓FA (% tract)	*r*	↑MD (% tract)	*r*	↑RD (% tract)	*r*	↑AX (% tract)	*r*	↓FA (% tract)	*r*	↑MD (% tract)	*r*	↑RD (% tract)	*r*	↑AX (% tract)	*r*
1	L-ATR	59.2	0.41	89.2	0.55	85.6	0.54	76.0	0.49	1.3	0.15	16.1	0.24	13.4	0.21	9.5	0.23
2	R-ATR	62.2	0.41	87.7	0.53	85.9	0.52	73.4	0.47	0.0	0.12	11.2	0.20	5.6	0.18	6.1	0.21
3	CC-FNR	93.4	0.60	97.7	0.61	98.3	0.64	56.7	0.40	3.4	0.20	32.7	0.29	27.9	0.27	14.1	0.23
4	CC-OCC	17.7	0.48	34.7	0.51	36.4	0.53	7.0	0.34	0.7	0.18	8.6	0.25	5.0	0.24	9.0	0.23
5	CC-PAR	37.6	0.49	59.9	0.52	64.4	0.55	15.4	0.35	10.6	0.21	43.1	0.30	43.1	0.28	24.4	0.26
6	CC-POCG	61.7	0.50	67.0	0.53	75.8	0.55	17.5	0.35	9.5	0.22	23.2	0.28	32.0	0.28	9.8	0.24
7	CC-PRCG	59.0	0.50	68.2	0.52	77.2	0.55	15.9	0.34	7.5	0.22	19.9	0.28	27.8	0.27	9.0	0.24
8	CC-TEM	25.6	0.49	53.7	0.52	50.0	0.54	34.8	0.36	0.3	0.19	35.0	0.29	14.1	0.26	40.5	0.27
9	L-CGC	56.3	0.51	74.0	0.53	79.9	0.56	24.1	0.35	6.5	0.21	31.1	0.28	30.9	0.28	6.7	0.23
10	R-CGC	57.4	0.51	79.0	0.54	82.0	0.57	26.2	0.35	1.8	0.20	25.3	0.28	24.7	0.27	4.3	0.22
11	L-CST	2.7	0.51	40.2	0.52	13.3	0.54	40.0	0.37	1.2	0.19	4.4	0.26	4.5	0.25	8.2	0.22
12	R-CST	4.2	0.51	51.0	0.52	22.4	0.54	46.4	0.39	1.0	0.19	5.5	0.25	5.7	0.24	3.9	0.22
13	L-IFO	49.9	0.52	70.8	0.55	66.5	0.57	41.4	0.37	1.3	0.19	12.3	0.25	12.3	0.25	2.9	0.21
14	R-IFO	38.8	0.51	63.7	0.54	59.0	0.56	34.8	0.36	0.6	0.19	7.1	0.25	7.4	0.24	1.2	0.20
15	L-ILF	14.2	0.47	53.7	0.52	44.3	0.53	34.8	0.37	0.7	0.18	24.2	0.27	16.6	0.25	11.5	0.23
16	R-ILF	18.9	0.47	49.1	0.50	45.4	0.52	17.5	0.34	0.2	0.17	17.2	0.26	6.9	0.23	11.3	0.23
17	L-PHC	27.6	0.48	69.4	0.51	62.6	0.53	43.2	0.35	14.1	0.19	33.1	0.27	39.1	0.25	9.4	0.23
18	R-PHC	23.4	0.47	67.6	0.51	59.8	0.53	33.2	0.35	9.9	0.19	30.4	0.27	35.7	0.26	9.9	0.23
19	L-SLF	41.3	0.48	83.0	0.53	75.0	0.54	29.9	0.35	2.3	0.18	34.5	0.28	17.0	0.25	16.1	0.24
20	L-UNC	94.6	0.53	97.9	0.56	99.5	0.58	70.4	0.38	4.2	0.20	15.0	0.27	20.8	0.25	5.7	0.23
21	R-UNC	81.3	0.52	97.8	0.56	98.7	0.58	61.6	0.38	1.5	0.19	8.8	0.27	8.2	0.25	5.2	0.23

bvFTD participants showed severe deficits, most frequently detected as decreases in FA (↓) (FDR critical *P* = 0.024), increases in MD (↑) (FDR critical *P* = 0.035), RD (FDR critical *P* = 0.034) and AX (FDR critical *P* = 0.019). Most affected tracts in bvFTD were the L-UNC, R-UNC, CC-FNR, L-ATR, R-ATR, L-CGC, R-CGC and L-SLF (Fig. 2). EOAD participants showed significant decreases in FA (FDR critical *P* = 1.5 × 10^−3^), increases in MD (FDR critical *P* = 0.01), RD (FDR critical *P* = 9.2 × 10^−3^) and AX (FDR critical *P* = 5.2 × 10^−3^). The L-PHC, R-PHC and CC-PAR stood out as most affected (Fig. 3). The percentage of tracts that passed FDR threshold is listed and total effect size (*r*) computed across each fiber

Within fibers, there was differential sensitivity of each of these metrics across the length of the fibers but overall, MD and RD measures detected most widespread changes in the white matter of bvFTD patients. While some exceptions are noted, the frontal and/or temporal tract components generally appeared the most impacted, with fewer changes observed in more posterior regions. Beginning with commissural fibers, we observed the most significant disruptions in the frontal fibers (CC-FNR, over 99 % fibers affected), and the percentage of fibers affected reduced moving posteriorly over the length of the corpus callosum (CC). The genu of the CC, which primarily innervates the frontal lobes, is one of the late myelinating white matter regions and is vulnerable to age-related alterations characterized by altered diffusion anisotropy (Kochunov et al. 2007). Regarding the long association fibers, over 99 % of fibers in the bilateral uncinate (L-UNC and R-UNC), which connects anterior temporal and orbitofrontal regions, were affected. Changes were observed in both the left superior longitudinal and bilateral inferior fronto-occipital fasiculi (L-SLF, L-IFO and R-IFO), which provide communication between frontal, parietal, temporal, and occipital lobes. Least affected of the long association fibers were the bilateral inferior longitudinal fasiculi (L-ILF and R-ILF), connecting the temporal and occipital cortices. Changes were also observed in the major associations fibers of the limbic system, including both the dorsal (R-CGC and L-CGC) and to a lesser extent ventral (R-PHC and L-PHC) aspects of the cingulum (CGC). Disconnections in the fibers connecting the limbic association cortex (i.e., parahippocampal gyrus, temporal pole and insula) could lead to impaired emotional response, often seen in patients with bvFTD (Geschwind 1965a; Catani and ffytche 2005). Furthermore, the anterior thalamic radiation, providing transmission between the thalamus and frontal lobe, showed significant changes. Among the least affected fibers were the bilateral corticospinal tracts.

### EOAD vs. controls

Our tract analyses detected decreases in FA (FDR critical *P* = 1.5 × 10^−3^) and increases in MD (FDR critical *P* = 0.01), RD (FDR critical *P* = 9.2 × 10^−3^) and AX (FDR critical *P* = 9.2 × 10^−3^) in all 21 bundles in EOAD, relative to controls (Figs. 1 and 3). As seen in the bvFTD vs. healthy controls comparisons, these DTI changes across the 4 measures did not occur simultaneously in a common set of fibers. For most white matter bundles, MD and RD increased in EAOD vs. controls across the same segments, while FA stayed relatively constant (or decreased) and AX increased (or stayed relatively unchanged).

The overall proportion of fibers affected was numerically smaller in EOAD than in bvFTD (Table 3). Even so, more local lesions may affect cognition and behavior, even if they are not as widespread as the white matter alterations mapped in bvFTD. Among the commissural fibers, the parietal segment of the CC was heavily affected (CC-PAR). These parietal fibers belong to higher-order association areas that greatly expanded in humans, allowing cross-modal association without the involvements of the limbic system (Geschwind 1965a). Lesions to this association cortex may disconnect primary receptive or motor areas from other areas on the cortex (Geschwind 1965a; Catani and ffytche 2005). Furthermore, the association fibers of the limbic system were also greatly disrupted, particularly in the ventral aspect of the CGC (L-PHC and R-PHC) beneath the hippocampus, followed by the dorsal aspect of the CGC (L-CGC and to a lesser extent, R-CGC). Disconnection of the limbic structures may lead to failure to evoke memories and affective responses when a stimulus is presented in that modality (Geschwind 1965a; Catani and ffytche 2005).

The long association fibers between cortical lobes also showed alterations. In general, both the bilateral uncinate (R-UNC and L-UNC) and inferior fronto-occipital fasciculi (R-IFO and L-IFO) showed alterations in the anterior portions of the tract, while more posterior aspects of the bundles were more affected in the left superior longitudinal fasciculi (L-SLF). There was some variability in this generalization depending on the specific DTI metric examined. Progressive disconnections may arise with disruptions to fibers passing through the association cortices that act as a relay station between different regions of the brain (Geschwind 1965a; Catani and ffytche 2005). Finally, the anterior segment of the anterior thalamic radiation (L-ATR and to a lesser extent, R-ATR) was affected. Least affected across all bundles were the corticospinal tracts (R-CST and L-CST).

### BvFTD vs. EOAD

BvFTD patients generally showed greater white matter abnormalities than EOAD patients. In our point-wise analyses, most DTI measures indicated decreases in FA in bvFTD (FDR critical *P* = 0.012), increases in MD (FDR critical *P* = 0.014), RD (FDR critical *P* = 0.015) and AX (FDR critical *P* = 4.8 × 10^−3^) – but again, across distinct segments of the fiber bundles (Online Resource 1). Alterations were found in the frontal connections of bvFTD patients in a similar pattern of disruption as seen in the EAOD vs. healthy controls comparisons (Fig. 4). These are specific atrophic patterns that differentiate bvFTD from AD in general; patients with bvFTD show prominent atrophy in the frontal lobe early in the disease, while AD patients tend to show these atrophic patterns only at later stages of disease progression (Thompson et al. 2003). In a few instances, we found that EOAD patients had more disrupted white matter integrity, compared to bvFTD, in regions of the brain known to be vulnerable to disease, including the temporal and parietal CC, and the ventral regions of the CGC. FA detected few alterations in the inferior segments of the corticospinal tracts in EOAD patients.

### Ranking DTI measures for detecting white matter abnormalities

For each DTI measure we averaged the fraction of significantly affected fibers (mean % tract) detected in the comparisons of all 21 tracts. MD detected the largest proportion of alterations in bvFTD vs. controls (mean 69 % tract, *r* = 0.53), followed by RD (mean 66 % tract, *r* = 0.55), FA (mean 44 % tract, *r* = 0.49) and AX (mean 38 % tract, *r* = 0.37) (Fig. 5). Effect sizes were similarly averaged across all DTI measures and results are illustrated in the Online Resource 2 figure. As changes in MD and RD were far more extensive than those detected by AX and FA, we averaged the % tract changes between RD and MD and ranked the fiber bundles with most altered fiber integrity metrics. Tracts with greatest deficiencies in bvFTD, compared to controls, in order from highest to lowest were: L-UNC, R-UNC, CC-FNR, L-ATR, R-ATR, R-CGC, L-SLF, L-CGC, CC-PRCG, CC-POCG, L-IFO, L-PHC, R-PHC, CC-PAR, R-IFO, CC-TEM, L-ILF, R-ILF, R-CST, CC-OCC and L-CST. Effect sizes were, in most cases, equivalently high and approximately reflected the rankings indicated by the % tract changes.

In EOAD vs. controls, MD (mean 21 % tract, *r* = 0.26) then RD (mean 19 % tract, *r* = 0.25) detected greatest white matter alterations across all 21 bundles, followed by AX (mean 10 % tract, *r* = 0.23) and FA (mean 4 % tract, *r* = 0.19). Based on the ranked % tract changes averaged across MD and RD, the most significantly affected individual tracts were as follows (ranked from high to low): CC-PAR, L-PHC, R-PHC, L-CGC, CC-FNR, CC-POCG, L-SLF, R-CGC, CC-TEM, CC-PRCG, L-ILF, L-UNC, L-ATR, L-IFO, R-ILF, R-UNC, R-ATR, R-IFO, CC-OCC, R-CST and L-CST.

When comparing bvFTD and EOAD patients, DTI measures RD (mean 24 % tract, *r* = 0.42) then MD (mean 23 % tract, *r* = 0.39) detected most % tract changes, followed by FA (mean 18 % tract, *r* = 0.41) and AX (mean 9 % tract, *r* = 0.26). From % tract changes detected by RD and MD, we obtained the following high to low ranking of the white matter tracts with greatest alterations: L-UNC, CC-FNR, L-ATR, R-ATR, R-UNC, L-IFO, R-IFO, R-CGC, L-CGC, R-CST, L-SLF, L-ILF, CC-POCG, L-CST, CC-PRCG, R-ILF, CC-TEM, L-PHC, CC-OCC, R-PHC and CC-PAR. These rankings indicate a more impaired white matter structure in bvFTD vs. EOAD. Meanwhile, EOAD patients showed greater abnormalities than bvFTD across a small fraction of fibers mostly in the R-CST and L-CST detected by FA with small effect sizes (5.6-2.8 % tract, *r* = 0.1), CC-TEM (2.8 % tract, *r* = 0.08) and CC-PAR (2.7 % tract, *r* = 0.1) detected by AX.

### Associations with cognition

To relate white matter deficits to a global measure of cognitive function, we tested for correlations with Mini Mental State Examination (MMSE) scores. In our point-wise analyses, MMSE scores decreased with increasing MD (FDR critical *P* = 2.0 × 10^−4^), RD (FDR critical *P* = 1.6 × 10^−4^) and AX (FDR critical *P* = 1.3 × 10^−5^) measures. The changes in the parietal segment of the callosal fibers stood out the most, but were detected with relatively small effect sizes (*r* = 0.1–0.2), as were the rest of the changes (Online Resource 3). These smaller effect sizes could be explained by the fact that MMSE scores may be less sensitive at detecting cognitive decline in FTD as compared to AD populations, or perhaps larger studies are needed to pick up associations between changes in the white matter and cognitive scores.

## Discussion

In this study, we applied an advanced fiber clustering workflow that extracts 3D anatomical models of white matter tracts to differentiate between patients with bvFTD in contrast with age-matched EOAD patients and healthy elderly. Our analyses provide comprehensive maps of fiber properties and indicate widespread white matter alterations across 21 distinct fiber bundles in bvFTD – in brain regions implicated in the socioemotional deficits seen in these patients. Most distinctive deficiencies in bvFTD, compared to controls, were located in the uncinate fasciculus bilaterally (UNC), frontal segment of the corpus callosum (CC-FNT), anterior thalamic radiation fibers bilaterally (ATR), left and right cingulum bundles (CGC), left superior longitudinal fasciculus (SLF) and inferior fronto-occipital fasciculus (IFO) bilaterally. This white matter profile was also identified when bvFTD patients were compared to EOAD, so these tracts might be involved in a relatively specific network of pathologies in the bvFTD spectrum. EOAD patients, although less impaired than bvFTD, also showed characteristic alterations linked with cognitive decline and areas with neurodegeneration, prominently in posterior parietal white matter fibers including the parietal callosal fibers (CC-PAR) and parahippocampal cingulum (PHC) bilaterally. Furthermore, we provide detailed maps of deficient white matter structure and ranked DTI measures based on their sensitivity to alterations across all white matter tracts of interest.

White matter pathways degenerate in the dementias leading to a progressive breakdown in an anatomically and functionally integrated system (Acosta-Cabronero et al. 2010). The white matter pathways analyzed in this study are plausible candidates for a characteristic dysfunctional network in both forms of dementia. First, relevant to the features of bvFTD – the UNC fibers, most affected in these patients, are involved in evaluation of affective and inter-personal signals (von der Heide et al. 2013), risk taking behaviors (Linke et al. 2013) and the modulation of inhibition (Hornberger et al. 2012). Next most affected were the CC and CGC bundle, implicated in the pathogenesis of executive dysfunction and obsessive-compulsive disorder (Bora et al. 2011, Linke et al. 2013). The CGC bundle innervates the cingulate cortex – a key component of the ‘Salience Network’ involved in the pathogenesis of bvFTD (Mahonney et al. 2012; Zhou et al. 2012). Moreover, ATR fibers are involved in attention and executive function (Andreasen et al. 1996; Schmahmann and Pandya 2008). The involvement of anterior parts of the IFO (especially in the left hemisphere), SLF, PHC and inferior longitudinal fasciculus (ILF) in bvFTD is less understood but might be linked with the evolution of pathology across white matter tracts in a network targeted by neurodegeneration. The least yet still significantly affected white matter tracts in bvFTD patients were the corticospinal tracts (CST), previously shown to degenerate, to some extent, in frontotemporal lobar degeneration (associated with FTD clinical syndrome) with TDP-43 immunoreactive inclusions (Josephs et al. 2013). White matter changes in the CST have been previously shown in amyotrophic lateral sclerosis patients (Foerster et al. 2013; Kasper et al. 2014) – a form of motor neuron disease with clinicopathologic features that often overlap with FTD (Agosta et al. 2010).

In EOAD, the neural networks affected have not been thoroughly investigated, but the patterns of alteration align with known profiles of cortical degeneration in LOAD patients. White matter alterations in the PHC extend into the posterior CGC bundle – an area commonly implicated in AD and MCI. Deficiencies in these areas may further extend into the posterior temporo-parietal areas and may progress to subsequent degeneration of interhemispheric white matter connections passing through the caudal CC (Acosta-Cabronero et al. 2010). In line with this, recent studies have reported a remarkable homology between atrophy in AD patients (in their temporal-posterior cingulate, precuneus and temporo-parietal cortex) and the ‘Default Mode Network’ in functional MRI (fMRI) (Seeley et al. 2009). White matter connections between these structures were also significantly affected in the current analysis. Major cortico-cortico white matter pathways – the SLF, ILF, UNC and IFO, subserving association cortices – were predominantly affected in the left hemisphere as shown previously in AD and MCI (Pievani et al. 2010). Finally the ATR fibers, previously associated with severity of apathy in early AD, and the CST fibers were least affected in EOAD.

Prior studies have reported widespread gray matter loss in the cortices of EOAD patients (with relative hippocampal sparing) as the disease advances (Frisoni et al. 2007). This gray matter loss may lead to secondary atrophy in the CC (Thompson et al. 1998) and other white matter regions, as seen in our study. Frisoni and colleagues found prominent gray matter alterations in the occipital lobe (24 % and 25 % of the right and left hemispheres were affected) and parietal lobe (with 23 % loss in both hemispheres), as well as in 25 % or more of the neocortical areas spreading across the lobes. While gray matter deficiencies do not directly translate to white matter findings, the more subtle and focal white matter tract degeneration we detected here in EOAD may partly be due to differences in the cohorts assessed. The EOAD population in (Frisoni et al. 2007), showed memory disturbances exceeding episodic forgetfulness and may have been more severely impaired in other domains (language, praxis, visuospatial skills). Our EOAD patients had a higher average MMSE score (23.4) than their EOAD patients (19.8) and were much less cognitively impaired and appeared to be at an earlier stage of their disease. Nonetheless, most of their findings are in line with our study, especially the alterations detected in the parietal lobe. We found that 43 % of the fibers were altered in the parietal segment of the CC (which innervate the parietal lobe, among other cortical regions). Furthermore, we also found that around 24 % of the left hemisphere and 17 % of the right hemisphere ILF fibers – connecting the temporal to the occipital lobe, were affected. Other fiber bundles with >30 % deficiencies were the right and left PHC, the left CGC bundle and frontal segment of the CC. These tract alterations are consistent with the widespread alterations (Frisoni et al. 2007) found across the neocortex. Furthermore, in AD, cortical alterations, including amyloid deposition and neurofibrillary tangle formation, may precede detectable white matter deficiencies.

Our findings may reveal a rather symmetric landscape of white matter abnormalities in bvFTD and more left than right hemisphere dominant abnormalities in EOAD. Although the two forms of dementia may start in an asymmetric fashion, they both have bilateral involvement. The behavioral variant of FTD tends to involve greater right frontotemporal changes, but neuropathology reveals involvement of the left hemisphere as well. Our study may indicate that the asymmetry in bvFTD is not as prominent for white matter as it is for gray matter. Meanwhile, some prior investigations have reported more left than right atrophy in AD patients, although this is not universally found. Thompson and colleagues showed predominant atrophic deficits in the left hemisphere of AD as well as a faster rate of local gray matter loss in the same hemisphere (Thompson et al. 2003). A recent network analysis study in AD participants also showed more impaired network connections in the left hemisphere than in the right (Daianu et al. 2013). Given that the neuropathological hallmarks are similar in AD and EOAD, asymmetric involvement of the left hemisphere may occur in some people with AD, remains intriguing, and needs further investigation.

Turning to the methodological details of this work, FA is perhaps the most widely used diffusion MRI metric in studies of dementia, but recent studies suggest that metrics AX and RD might have more specificity for certain pathologies and may be more appropriate descriptors of neurodegeneration (Mahoney et al. 2014; Jones et al. 2013). Using FA as the only descriptor of axonal integrity assumes that degeneration leads to changes in a shape of an ellipsoid (Acosta-Cabronero et al. 2010). However, demyelination and axonal damage are driven by increases in RD and often a constant AX (Beaulieu 2002; Song et al. 2002; Mahoney et al. 2014). On the other hand, simultaneous increases in RD and decreases in AX can reflect fiber reorganization (Dubois et al. 2008). It was previously shown that decreases in AX are associated with axonal damage in mouse models of white matter injury (Sun et al. 2006; Kim et al. 2007). MD is the average amount of water diffusion in the tissue and depends on measures AX and RD, making it difficult to interpret microstructurally. Generally, increases in MD indicate higher rates of diffusion (Bennett et al. 2010). Changes in MD have been associated with neuroinflammation in schizophrenic patients explained by excessive extra-cellular volume (Pasternak et al. 2012). Although neuroinflammation is often reported as a key component in the etiology of neurodegenerative diseases in both FTD (Filiano et al. 2013) and AD (Weiner and Selkoe 2002; Krstic and Knuesel 2013), to our knowledge, it has not been directly linked to increases in MD. It is important to note, however, that in complex diseases that include demyelination, axonal damage and inflammation, it is yet unclear how accurately the standard DTI metrics relate to the specific pathologies (Budde et al. 2009). Other processes leading to white matter degeneration such as glial degeneration and destruction of neurofibrils (Acosta-Cabronero et al. 2010) can lead to tensor properties that are either not fully captured by changes in anisotropy or cannot be fully explained in DTI studies.

In our work, the DTI metrics MD followed by RD detected the most comprehensive extent of white matter alterations in both forms of dementia. In bvFTD vs. controls (Fig. 2), changes in FA and AX were found, but to a lesser extent: in these patients, RD and MD increased, FA decreased and AX stayed relatively constant or increased. On the other hand, in EOAD vs. controls (Fig. 3), we most often observed concurrent increases in MD and RD with increases in AX over a smaller proportion of fibers, while FA was least sensitive to changes, indicating, as previously reported (Acosta-Cabronero et al. 2010), that it may not be the optimal measure for studying white matter degeneration in AD.

It is often assumed that the primary diffusion tensor is aligned with white matter bundles and the average diffusion perpendicular to the fiber tracts (RD) is modulated by extracellular distance between axonal membranes and degree of myelination. Even so, other biological phenomena affect water diffusion in the brain (Acosta-Cabronero et al. 2010). Increases in membrane permeability, fiber reorganization, destruction of intracellular compartments (Acosta-Cabronero et al. 2010), may induce faster diffusion. This might explain why we observed significant increases in AX, concordant with MD and RD, yet minimal changes in anisotropy. These findings reinforce the concept that exploring multiple diffusion indices is important; changes in MD and RD may be more sensitive to some degenerative processes than changes in FA. Ongoing related work has been assessing the value of combining numerous DTI metrics, weighted by their ability to detect disease-specific changes, to better identify white matter alterations in dementia. In one such approach (Nir et al. 2015), we aimed to find the most helpful diffusion MRI metrics and brain regions to distinguish AD from normal aging, from a set of 17 diffusion MRI feature maps. In white matter areas near the cortex, metrics of neurite dispersion based on the NODDI model of diffusion (Zhang et al. 2012) tended to perform best, but in subcortical white matter areas where fiber pathways mix and cross, the best measures were those derived from the tensor distribution function (Leow et al. 2009). Even though all of the diffusion MRI measures described are somewhat correlated with each other, each captures the microstructure in a slightly different way.

A limitation of the present study is the DTI reconstruction used to recover fibers in the white matter structure. Unlike high angular resolution imaging (HARDI), DTI is not always able to accurately reconstruct crossing fibers within a single image voxel (Daianu et al. 2015b), as it can only recover the primary orientation of the fiber tracts. This can overlook some white matter fiber populations and might limit the detection of crossing white matter pathways with tractography. A further challenge was the accurate fiber reconstruction of the fornix – a key locus of damage in patients with bvFTD (Hornberger et al. 2012). The pathway trajectory of the fornix is hard to reconstruct due to its small thickness and partial voluming with CSF. Future studies with high magnetic field MRI, or with diffusion spectrum imaging, may better resolve crossing fibers.

## Electronic supplementary material

Below is the link to the electronic supplementary material.

ESM 1 (DOCX 877 kb)

## Abbreviations

AD: Alzheimer’s disease
ATR: Anterior thalamic radiation
AX: Axial diffusivity
bvFTD: Behavioral variant frontotemporal dementia
CC: Corpus callosum
CC-OCC: Corpus callosum, occipital lobe
CC-PAR: Corpus callosum, parietal lobe
CC-POCG: Corpus callosum, post-central gyrus
CC-PRCG: Corpus callosum, pre-central gyrus
CC-TEM: Corpus callosum, temporal lobe
CGC: Cingulum
CST: Corticospinal tract
DTI: Diffusion tensor imaging
DWI: Diffusion weighted imaging
EOAD: Early-onset Alzheimer’s disease
FA: Fractional anisotropy
FDR: False discovery rate
FTD: Frontotemporal dementia
IFO: Inferior fronto-occipital fasciculus
ILF: Inferior longitudinal fasciculus
LOAD: Late-onset Alzheimer’s disease
MCI: Mild cognitive impairment
MD: Mean diffusivity
MMSE: Mini Mental State Examination
MRI: Magnetic resonance imaging
PHC: Parahippocampal cingulum
RD: Radial diffusivity
ROI: Region of interest
SLF: Superior longitudinal fasciculus
TE: Echo time
TI: Inversion time
TR: Relaxation time
UNC: Uncinate
