# Supplementary material for: An advanced white matter tract analysis in frontotemporal dementia and early-onset Alzheimer’s disease
Source: Brain Imaging Behav. 2015 Oct 29;10(4):1038–53. doi: 10.1007/s11682-015-9458-5 (PMC5167220; doi:10.1007/s11682-015-9458-5)
Supplement: Supplementary file 1 — (DOCX 877 kb) [file 11682_2015_9458_MOESM1_ESM.docx]

**Article Title**: A white matter pathway atlas for behavioral frontotemporal dementia and early-onset Alzheimer’s disease

**Journal Name**: Brain Imaging and Behavior

**Author Names:** Madelaine Daianu, Mario F. Mendez, Vatche G. Baboyan, Yan Jin, Rebecca J. Melrose, Elvira E. Jimenez, Paul M. Thompson

**Affiliation:** Imaging Genetics Center, Mark & Mary Stevens Institute for Neuroimaging & Informatics, University of Southern California, Marina del Rey, CA, USA

**e-mail address of the corresponding author:** madelaine.daianu@ini.usc.edu

**Supplementary Information: Online Resources**

| **#** | **ROI** | **FTD vs. EOAD** | | | | | | | |
| --- | --- | --- | --- | --- | --- | --- | --- | --- | --- |
|  |  | **🡻**FA  (% tract) | ***r*** | **🡹**MD  (% tract) | ***r*** | **🡹**RD  (% tract) | ***r*** | **🡹**AX  (% tract) | ***r*** |
| **1** | L-ATR | 41.8 | 0.36 | 63.4 | 0.44 | 64.2 | 0.44 | 37.3 | 0.38 |
| **2** | R-ATR | 40.3 | 0.36 | 64.3 | 0.43 | 63.0 | 0.43 | 34.0 | 0.37 |
| **3** | CC-FNR | 74.4 | 0.50 | 68.4 | 0.45 | 77.9 | 0.50 | 12.8 | 0.27 |
| **4** | CC-OCC | 1.8 | 0.40 | 1.8 | 0.38 | 2.8 | 0.41 | 0.3 | 0.25 |
| **5** | CC-PAR | 2.4 | 0.42 | 1.0 | 0.39 | 1.6 | 0.42 | 0.4 | 0.25 |
| **6** | CC-POCG | 2.4 | 0.41 | 5.7 | 0.38 | 2.7 | 0.41 | 3.5 | 0.25 |
| **7** | CC-PRCG | 2.0 | 0.41 | 5.4 | 0.38 | 1.7 | 0.41 | 2.1 | 0.25 |
| **8** | CC-TEM | 6.1 | 0.41 | 2.2 | 0.38 | 4.0 | 0.41 | 0.5 | 0.25 |
| **9** | L-CGC | 16.1 | 0.42 | 14.9 | 0.39 | 18.6 | 0.43 | 2.1 | 0.25 |
| **10** | R-CGC | 16.4 | 0.41 | 16.3 | 0.39 | 20.3 | 0.42 | 2.5 | 0.25 |
| **11** | L-CST | 0.5 | 0.43 | 5.3 | 0.38 | 1.9 | 0.42 | 10.5 | 0.26 |
| **12** | R-CST | 0.8 | 0.42 | 13.6 | 0.38 | 5.6 | 0.41 | 15.1 | 0.28 |
| **13** | L-IFO | 30.2 | 0.44 | 40.1 | 0.41 | 38.7 | 0.44 | 9.7 | 0.28 |
| **14** | R-IFO | 22.5 | 0.42 | 28.5 | 0.40 | 29.9 | 0.43 | 6.1 | 0.26 |
| **15** | L-ILF | 3.4 | 0.40 | 9.0 | 0.38 | 7.6 | 0.41 | 2.4 | 0.25 |
| **16** | R-ILF | 5.6 | 0.39 | 2.6 | 0.37 | 4.1 | 0.40 | 0.1 | 0.24 |
| **17** | L-PHC | 1.8 | 0.40 | 2.6 | 0.37 | 3.1 | 0.40 | 1.4 | 0.24 |
| **18** | R-PHC | 0.2 | 0.40 | 2.3 | 0.37 | 1.7 | 0.40 | 3.0 | 0.24 |
| **19** | L-SLF | 12.3 | 0.41 | 6.0 | 0.37 | 11.1 | 0.41 | 0.7 | 0.23 |
| **20** | L-UNC | 53.3 | 0.44 | 79.2 | 0.42 | 82.7 | 0.46 | 24.8 | 0.28 |
| **21** | R-UNC | 48.4 | 0.43 | 57.3 | 0.41 | 59.6 | 0.44 | 16.5 | 0.27 |

**Online Resource 1.** Group comparisons between bvFTD and EOAD from a point-wise tract correspondence analysis for measures FA, MD, RD and AX based on a linear regression, controlling for age, sex and brain volume. bvFTD subjects were more severely affected than EOAD, predominantly in frontal and medial-frontal network. Here we list decreases (**🡻**) in FA (FDR critical *P*=0.012), increases (**🡹**) in MD (FDR critical *P*=0.014), RD (FDR critical *P*=0.015) and AX (FDR critical *P*=4.8x10^-3^). Note than these DTI changes did not always occur over an overlapping set of fibers. The percentage of tracts that passed FDR threshold is listed and the total effect size (*r*) computed across each fiber.


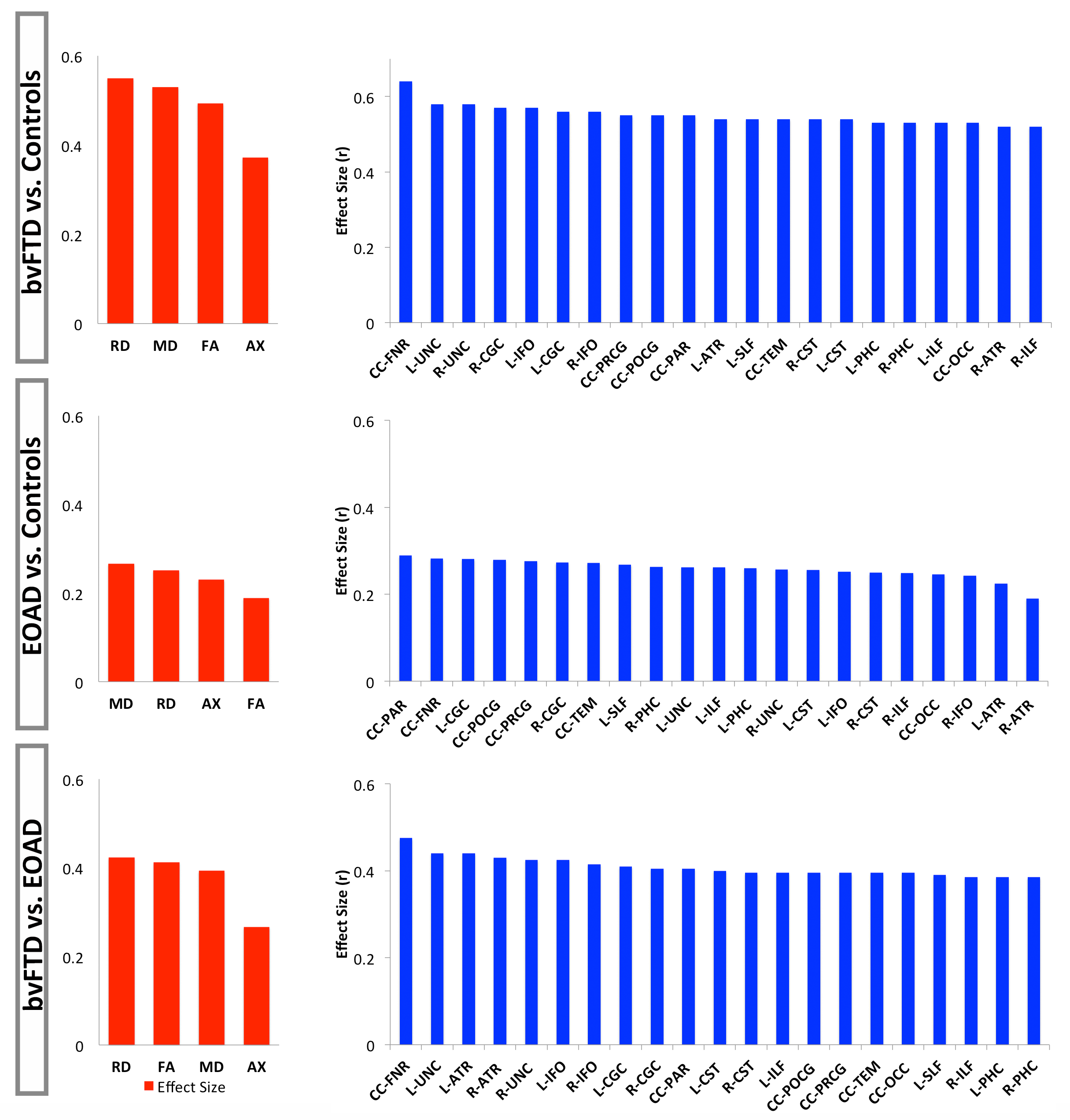


**Online Resource 2**. DTI measures ranked based on their ability to detect widespread white matter alterations as a function of effect size*.* The effect size for each DTI measure, FA, MD, RD and AX was averaged across changing segments of all 21 bundles. Most alterations were detected by measures RD and MD in the group comparisons (*red* bars). The effect sizes from RD and MD were averaged and the fibers with largest *r* were detected from high to low (*blue* bars).

| **#** | **ROI** | **Associations with MMSE scores across all participants** | | | | | |
| --- | --- | --- | --- | --- | --- | --- | --- |
|  |  | **🡹**MD  (% tract) | **r** | **🡹**RD  (% tract) | **r** | **🡹**AX  (% tract) | **r** |
| **1** | L-ATR | 0.2 | 0.1 | 0.3 | 0.1 | - | - |
| **3** | CC-FNR | 0.05 | 0.1 | 0.1 | 0.1 | - | - |
| **4** | CC-OCC | 0.2 | 0.1 | 0.2 | 0.1 | - | - |
| **5** | CC-PAR | 3.7 | 0.2 | 2.8 | 0.2 | 0.4 | 0.1 |
| **6** | CC-POCG | 0.2 | 0.1 | 0.1 | 0.1 | - | - |
| **7** | CC-PRCG | - | - | 0.7 | 0.1 | - | - |
| **8** | CC-TEM | 0.5 | 0.1 | 0.1 | 0.1 | 0.1 | 0.1 |
| **9** | L-CGC | 1.5 | 0.1 | 1.1 | 0.1 | - | - |
| **10** | R-CGC | 0.2 | 0.1 | 0.1 | 0.1 | - | - |
| **12** | R-CST | 0.1 | 0.1 | 0.1 | 0.1 | - | - |
| **13** | L-IFO | 0.1 | 0.1 | 0.1 | 0.1 | - | - |
| **15** | L-ILF | 0.6 | 0.1 | 0.9 | 0.1 | - | - |
| **16** | R-ILF | 0.8 | 0.1 | 0.2 | 0.1 | - | - |
| **17** | L-PHC | 0.1 | 0.1 | 0.1 | 0.1 | - | - |
| **18** | R-PHC | 0.1 | 0.1 | 0.1 | 0.1 | - | - |
| **19** | L-SLF | 0.8 | 0.2 | - | - | - | - |

**Online Resource 3.** MMSE associations with measures MD, RD and AD from the point-wise tract correspondence analyses across all 76 subjects combined. We ran a linear regression, controlling for age, sex, brain volume and disease status. MD, RD and AD increased (**🡹**) with decreasing MMSE scores. The percentage of tracts that passed FDR threshold is listed and the total effect size (*r*) computed across each fiber. No changes were detected by FA.
